# The disease burden attributable to tobacco use in China and its provinces from 1990−2023: an analysis from the Global Burden of Disease Study 2023

**DOI:** 10.1016/j.mmr.2026.100041

**Published:** 2026-05-23

**Authors:** Ling-Ling Yu, Fan-Shu Yan, Jin-Lei Qi, Li-Jun Wang, Mai-Geng Zhou, Peng Yin

**Affiliations:** National Center for Chronic and Noncommunicable Disease Control and Prevention, Chinese Center for Disease Control and Prevention, Beijing 100050, China

**Keywords:** Tobacco, Smoking, China, Global Burden of Diseases, Morality, Disability-adjusted life year

## Abstract

**Background:**

As the world’s leading producer and consumer of tobacco, China bears a significant health burden attributable to tobacco. This study aims to provide a precise, updated analysis of the demographic, temporal, and spatial dimensions of the tobacco-attributable disease burden across China.

**Methods:**

Using the Global Burden of Diseases, Injuries, and Risk Factors Study (GBD) 2023 comparative risk assessment framework and its hierarchical taxonomy, we analyzed tobacco use as a Level 2 (behavioral) risk. We estimated the national and provincial burdens of tobacco use in China from 1990 to 2023, quantifying deaths, years of life lost (YLLs), years lived with disability (YLDs), and disability-adjusted life years (DALYs) stratified by sex, age, cause, and the three Level 3 tobacco sub-categories: smoking, secondhand smoke (SHS), and chewing tobacco. Temporal trends were assessed via average annual percentage change (AAPC).

**Results:**

In 2023, tobacco use caused 2.38 million [95% uncertainty interval (UI) 2.02−2.83] deaths and 57.27 million (95% UI 48.28−60.82) DALYs, predominantly premature mortality [YLLs 49.45 million (95% UI 42.23−59.25)]. Direct smoking was the primary driver (2.01 million deaths), followed by SHS and chewing tobacco. A profound sex disparity existed in smoking burden, with the male age-standardized mortality rate (ASMR; 167.78/100,000) being 9.5-time higher than that of females, although females bore a greater SHS-attributable burden. From 1990 to 2023, absolute deaths and DALYs increased, but their age-standardized rates (ASRs) significantly declined (AAPC for mortality: −2.34%). The burden is concentrated in older adults, with the disease spectrum shifting from respiratory infections in youth to chronic diseases such as heart disease and lung cancer later in life. Geographically, northern provinces had the highest burden, whereas coastal areas had the lowest burden.

**Conclusions:**

Despite declining ASRs, China’s tobacco attributable burden is concentrated among males, older adults, and northern provinces. In the context of rapid aging and the long-term harmful effects of tobacco smoking, more stringent and targeted policies should be implemented to address the challenges associated with tobacco use in China.

## Background

Tobacco use remains the leading preventable cause of death worldwide [1]. As the world’s largest producer and consumer of tobacco, China carries more than 300 million smokers (nearly one‑third of the world’s total) and over half of its adult male population actively smoked in 2018 [1], [2]. The problem extends far beyond active smokers, with more than 700 million nonsmokers, including approximately 180 million children, who are regularly exposed to secondhand smoke (SHS) [3]. This exposure leads to an estimated 100,000 deaths per year, underscoring that SHS remains a critical public health crisis [1]. China has implemented various tobacco control measures in alignment with the World Health Organization (WHO) Framework Convention on Tobacco Control (FCTC) [4], the world’s first public health treaty, which has been a key driver in reducing tobacco consumption globally, and has intensified tobacco control through pricing, taxation, and legislation to actively promote the development of smoke-free environments and strengthen supervision and law enforcement in public places [5], [6]. Following these policy proposals and implementations, representative surveys have confirmed a consecutive annual decline in China’s smoking rates [2], [7], [8].

Accumulating evidence underscores the profound health impacts of tobacco use. Ten‑year cross‑sectional surveys revealed a nonlinear, inverse correlation between chronic disease risk and the age of smoking initiation, suggesting that earlier smoking is associated with greater long‑term morbidity [9]. Furthermore, prospective data from the China Kadoorie Biobank, for instance, indicate that smoking significantly elevates the risk of 22 causes of death and 56 individual diseases spanning all major organ systems, along with increasing the frequency and duration of hospitalizations [10]. Global Burden of Diseases, Injuries, and Risk Factors Study (GBD) 2021 revealed that over the past three decades, the age-standardized mortality rate (ASMR) for stroke attributable to tobacco in China has tended to decrease, whereas China has ranked fourth globally in the number of disability-adjusted life years (DALYs) from multidrug-resistant tuberculosis attributable to smoking [11], [12].

Despite these advancements and the growing body of evidence on the health impacts of tobacco, a critical gap remains in quantifying the comprehensive disease burden attributable to all forms of tobacco use [13]. Existing research has been limited, often focusing on a narrow spectrum of diseases, such as cancer [14], cardiovascular disease [15], and chronic respiratory diseases [16], [17], or relying on outdated cohort and cross-sectional data that lack geographic variations [9], [10], [18]. Consequently, the absence of a detailed and contemporary assessment of the national and subnational burden, which encompasses active smoking, SHS, and smokeless tobacco (with a prevalence under 1%) [2], hinders the development of precisely targeted and efficient control policies.

This study aimed to determine the overall tobacco-attributable disease burden in China, as well as by sex, age group, and subregion. A precise understanding of the demographic, temporal, and spatial dimensions of burden attributable to tobacco is essential for setting targeted policies, identifying high-risk regions for intervention, and guiding efficient resource allocation to improve health outcomes and promote equity.

## Methods

### Overview

The GBD 2023 provides systematic estimates from 1990 to 2023 for the incidence, prevalence, years lived with disability (YLDs), years of life lost (YLLs), and DALYs for 375 diseases and injuries, all organized within a four-level cause hierarchy [19]. In addition, the study quantified the burden attributable to 88 behavioral, environmental, occupational, and metabolic risk factors across 204 countries and territories [20]. In the GBD hierarchical risk taxonomy, these risk factors are organized into three primary domains: behavioral, environmental, and occupational and metabolic. Tobacco use is classified as a Level 2 risk factor within the behavioral domain. For this specific analysis, the burden attributable to tobacco was disaggregated into three Level 3 subcategories: smoking, SHS, and chewing tobacco. In line with best practices for reporting, this study adheres to the Guidelines for Accurate and Transparent Health Estimates Reporting (GATHER) to ensure full transparency and reproducibility of its findings [21].

Our analysis follows the GBD 2023 comparative risk assessment (CRA) framework and its hierarchical taxonomy. Attributable burden estimates, uncertainty analysis, and temporal trend analysis are detailed in **Additional file 1: Methods**.

### Definitions

All definitions were consistent with the cornerstone of the GBD 2023 [20]. The overarching concept “attributable burden” refers to the reduction in the current disease burden that would have occurred if past population exposure to a risk factor had been lowered to a theoretical minimum risk exposure level (TMREL) [20]. Current smoking was defined as the daily or occasional use of any combustible tobacco product, including manufactured cigarettes, hand-rolled cigarettes, pipes, cigars, shisha, and other locally smoked tobacco products. Smokeless tobacco, electronic cigarettes (e-cigarettes), and heated tobacco products were explicitly excluded as their long-term population health risks are not yet fully quantified within the GBD framework, and the primary aim of this study was to assess the burden attributable to conventional combustible tobacco use, which remains the dominant form of consumption and has well-established risk estimates. SHS was operationalized as current exposure to tobacco smoke at home or in the workplace. Only individuals classified as nonsmokers, defined as those not identified as daily smokers, including former and occasional smokers, were considered susceptible to SHS exposure. Household composition served as a proxy for domestic exposure, whereby all individuals living with a daily smoker were assumed to be exposed. For workplace exposure, survey data were used to estimate the proportion of the population exposed to SHS at work. This assessment was applied to both children and adults. Current chewing tobacco use is defined as the use of any smokeless tobacco product, with “current” indicating use within the past 30 d where data permitted, or according to the closest available survey definition. This included products of any use frequency (daily or nondaily) and covered local preparations such as betel quid.

### Data source and process

According to the GBD study 2023, the estimation of the tobacco-attributable disease burden relies on four inputs: 1) exposure; 2) relative risk (*RR*) of each health outcome associated with the risk factor; 3) the TMREL; 4) deaths and burden for each of the health outcomes with which a risk factor is associated. The TMREL and deaths and burden for each health outcome associated with a risk factor are derived within the GBD study, and our data acquisition focused on exposure and *RR*s [20].

Data for tobacco exposure and *RR* were obtained primarily from nationally and provincially representative surveys, censuses, polls, and cohorts conducted in China since 1982 (**Additional file 1:** Table S1). To increase the robustness and comparability of the exposure data, the meta-regression-Bayesian, regularized, trimmed (MR-BRT) methodology was applied to adjust for systematic measurement bias and to disaggregate data by age and sex. These adjusted data were then modeled via spatiotemporal Gaussian process regression (ST-GPR) to generate continuous exposure estimates by age, sex, location, and year from 1990 to 2023. Exposure distributions across individuals were estimated by modeling the standard deviation and fitting parametric distributions. All steps described above represent the proprietary modeling framework of the GBD 2023 study. For this study, we utilized the direct output of this framework, specifically the final, age-standardized, tobacco-attributable burden estimates at the provincial level, for all subsequent spatiotemporal and stratified analyses.

The *RR* estimates were consolidated from the published literature, encompassing cohort, case-control, and cross-sectional studies, and evaluating 676 risk-outcome pairs across all risk factors. The burden from direct smoking encompasses numerous cancers, cardiovascular, respiratory, and metabolic diseases. SHS is linked to conditions including several cancers, cardiovascular diseases, chronic obstructive pulmonary disease (COPD), diabetes, and newly established links to asthma. Chewing tobacco was associated with upper aerodigestive tract cancers and stroke (**Additional file 1:** Table S2). These associations are based on *RR*s modeled within the GBD framework, with the effects largely mediated through exposure to SHS. For a subset of pairs, the burden of proof (BPRF) meta-regression approach was applied, using ensemble spline models to capture nonlinear dose-response relationships, trim outliers, adjust for study design covariates, and quantify between-study heterogeneity. This generates a conservative BPRF, which is translated into risk-outcome scores and star ratings indicating evidence strength. For tobacco use, the uncertainty interval (UI) for *RR*s excludes between-study heterogeneity due to policy interpretation considerations. All data sources are available through the GBD 2023 Sources Tool (https://ghdx.healthdata.org/gbd-2023/sources).

### Statistical analysis

Focusing on burden estimates rather than prevalence trends, this study aimed to quantify the direct health impact of tobacco use in China. We extracted estimates for the disease burden attributable to overall tobacco use and its three subcategories in China. The primary metrics included the absolute counts, age-standardized rates (ASRs), and percentage changes for deaths, DALYs, YLLs, and YLDs from 1990 to 2023, all stratified by sex and presented with 95% UI. ASRs were calculated by using the GBD world standard population. The total percentage change represents the net difference between the estimated ASRs in the start and end years (1990 and 2023).

For the year 2023, estimates of deaths and DALYs were further stratified by 5-year age groups, encompassing a total of 17 age groups from <20 years to ＞95 years. To delineate the disease spectrum across the lifespan, we also obtained the specific Level 3 causes of deaths and DALYs attributable to tobacco use for each age group. A complete list of these causes and corresponding International Classification of Diseases (ICD) codes is provided in **Additional file 1:** Table S3. Furthermore, a subnational analysis was conducted to investigate the geographical variation in age-standardized mortality and DALY rates across 31 provincial-level administrative units in China. Joinpoint regression was performed with Joinpoint software (version 5.4.0), and other statistical analyses were conducted with R (version 4.5.3).

## Results

### Tobacco-attributable burden in China from 1990−2023

In 2023, tobacco use was responsible for a substantial health burden in China, resulting in an estimated 2.38 million (95% UI 2.02−2.83) deaths and 57.27 million (95% UI 48.28−60.82) DALYs (Table 1). The composition of this burden was dominated by premature mortality, which contributed 49.45 million (95% UI 42.23−59.25) of YLLs and 86.3% of total DALYs, compared with 7.81 million (95% UI 5.39−10.33) resulting from YLDs. The attribution of this burden revealed a clear hierarchy among tobacco products. Smoking was the predominant driver, accounting for the vast majority of both deaths [2.01 (95% UI 1.66–2.47) million; 84.5%] and DALYs [49.61 (95% UI 40.83–60.80) million; 86.6%]. SHS constituted the second-largest contributor, underscoring its significant public health impact (Table 1).

**Table 1:** Counts and age-standardized rates (ASRs) of deaths, DALYs, YLDs, and YLLs attributable to tobacco use, by sex and tobacco use type, in 2023.

**Tobacco type**	**Deaths**		**DALYs**		**YLDs**		**YLLs**	
	**Number (million, 95% UI)**	**ASR (/100,000, 95% UI)**	**Number (million, 95% UI)**	**ASR (/100,000, 95% UI)**	**Number (million, 95% UI)**	**ASR (/100,000, 95% UI)**	**Number (million, 95% UI)**	**ASR (/100,000, 95% UI)**
Tobacco								
Both	2.38 (2.02−2.83)	105.60 (89.62−125.98)	57.27 (48.28−60.82)	2539.78 (2148.81−2999.66)	7.81 (5.39−10.33)	360.75 (247.64−480.63)	49.45 (42.23−59.25)	2179.03 (1860.35−2594.68)
Male	1.91 (1.60−2.28)	180.46 (151.61−215.11)	47.57 (39.31−56.97)	4344.20 (3591.27−5171.60)	6.14 (4.17−8.26)	574.97 (390.50−776.47)	41.43 (34.51−50.04)	3769.23 (3133.18−4502.69)
Female	0.47 (0.37−0.60)	39.24 (31.24−50.49)	9.69 (7.87−11.97)	836.98 (677.19−1018.95)	1.67 (1.18−2.20)	151.58 (107.56−200.57)	8.02 (6.28−9.83)	685.40 (553.75−851.20)
Smoking								
Both	2.01 (1.66−2.47)	88.14 (72.41−108.05)	49.61 (40.83−60.80)	2172.21 (1783.48−2659.17)	6.59 (4.31−8.95)	299.25 (194.70−408.33)	43.02 (35.61−52.22)	1872.96 (1543.98−2274.28)
Male	1.80 (1.49−2.20)	167.78 (140.13−204.47)	45.18 (37.07−54.83)	4088.25 (3358.16−4968.24)	5.81 (3.86−7.87)	538.22 (356.33−733.88)	39.37 (32.43−47.59)	3550.03 (2930.96−4284.45)
Female	0.21 (0.14−0.31)	17.74 (11.81−25.74)	4.43 (2.99−6.34)	366.85 (245.77−524.63)	0.78 (0.43−1.20)	66.07 (37.20−103,79)	3.65 (2.51−5.24)	300.77 (206.94−428.64)
Secondhand smoke								
Both	0.49 (0.37−0.63)	22.51 (17.12−29.01)	10.14 (7.95−12.56)	475.84 (372.83−589.49)	1.43 (0.95−1.92)	70.70 (46.82−94.01)	8.67 (6.73−10.93)	405.14 (320.29−510.70)
Male	0.21 (0.16−0.27)	21.51 (15.87−27.89)	4.46 (3.37−5.73)	444.16 (334.79−568.08)	0.50 (0.32−0.68)	52.02 (32.66−70.40)	3.96 (2.96−5.12)	392.14 (293.22−505.88)
Female	0.28 (0.21−0.36)	23.28 (17.74−30.11)	5.68 (4.53−7.04)	504.57 (404.92−622.50)	0.94 (0.64−1.22)	88.88 (60.59−116.66)	4.74 (3.70−6.01)	415.70 (327.06−523.06)
Chewing tobacco								
Both	0.009 (0.003−0.019)	0.40 (0.13−0.86)	0.22 (0.08−0.48)	9.80 (3.39−21.63)	0.018 (0.006−0.046)	0.86 (0.29−2.20)	0.199 (0.066−0.427)	8.95 (3.16−19.62)
Male	0.007 (0.002−0.015)	0.65 (0.22−1.40)	0.18 (0.06−0.38)	16.24 (5.82−35.39)	0.013 (0.004−0.032)	1.20 (0.41−3.06)	0.163 (0.059−0.356)	15.04 (5.44−32.77)
Female	0.002 (0.0006−0.005)	0.17 (0.05−0.38)	0.04 (0.01−0.10)	3.59 (1.10−8.41)	0.005 (0.002−0.014)	0.52 (0.17−1.36)	0.036 (0.011−0.083)	3.08 (0.95−7.08)

DALYs. Disability-adjusted life years; YLLs. Years of life lost; YLDs. Years lived with disability

A profound sex disparity characterized the tobacco-attributable burden. The burden on males was disproportionately higher, with 1.80 million (95% UI 1.49−2.20) deaths and 45.18 million (95% UI 37.07−54.83) DALYs attributable to smoking alone, constituting 94.2% and 95.0% of the total tobacco-attributable burden of males, respectively. This disparity was further highlighted by the ASMR, which was 167.78/100,000 (95% UI 140.13−204.47) in males, a rate 9.5 times as high as that in females (17.74/100,000) (Table 1).

**Fig. 1:**
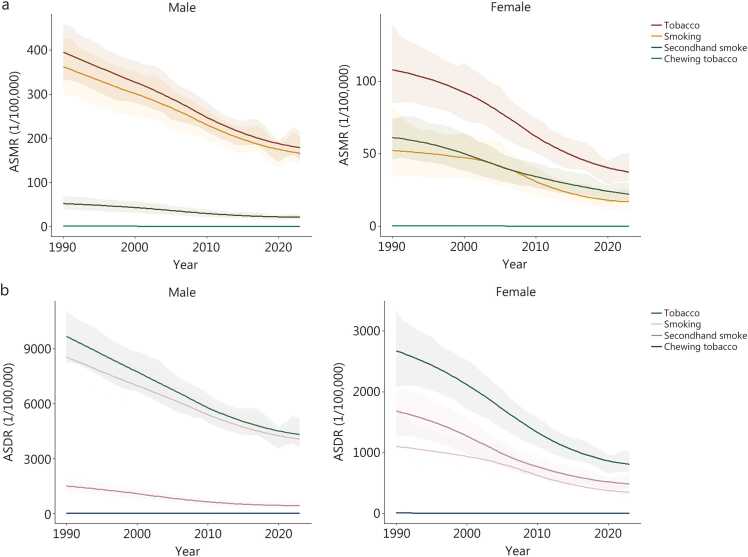
Trends in ASMR (**a**) and ASDR (**b**) attributable to tobacco use, by sex and tobacco type from 1990 to 2023. ASMR. Age-standardized mortality rate; ASDR. Age-standardized disability-adjusted life year rate.

From 1990 to 2023, the absolute number of deaths and DALYs attributable to tobacco increased, whereas their ASRs exhibited a modest decline of −0.55/100,000 (95% UI −0.63 to −0.45) and −0.58/100,000 (95% UI −0.64 to −0.50), respectively (**Additional file 1:** Table S4). This pattern of rising counts alongside falling rates was consistent across both sexes and all tobacco use categories (smoking, SHS, and chewing tobacco) (Fig. 1). The decline in risk at the population level is further underscored by a significant and steady average annual percentage change (AAPC) of −2.34% (95% CI −2.74 to −1.94; *P*<0.001) for deaths and −2.53% (95% CI −2.91 to −2.14; *P*<0.001) for DALYs over the 33-year period (**Additional file 1:** Table S5).

Throughout the study period, the ASMR and age-standardized disability-adjusted life year rate (ASDR) attributable to tobacco exhibited a general downward trend, with the burden consistently being greater in males than in females (Fig. 1). In females, SHS accounted for a larger proportion of the health burden (by both the ASMR and ADSR) than did direct smoking, which was opposite to that observed in males. The contribution of chewing tobacco remained minimal in both sexes and showed little variation over time in China (Fig. 1).

### Age distribution of burden attributable to tobacco use

**Fig. 2:**
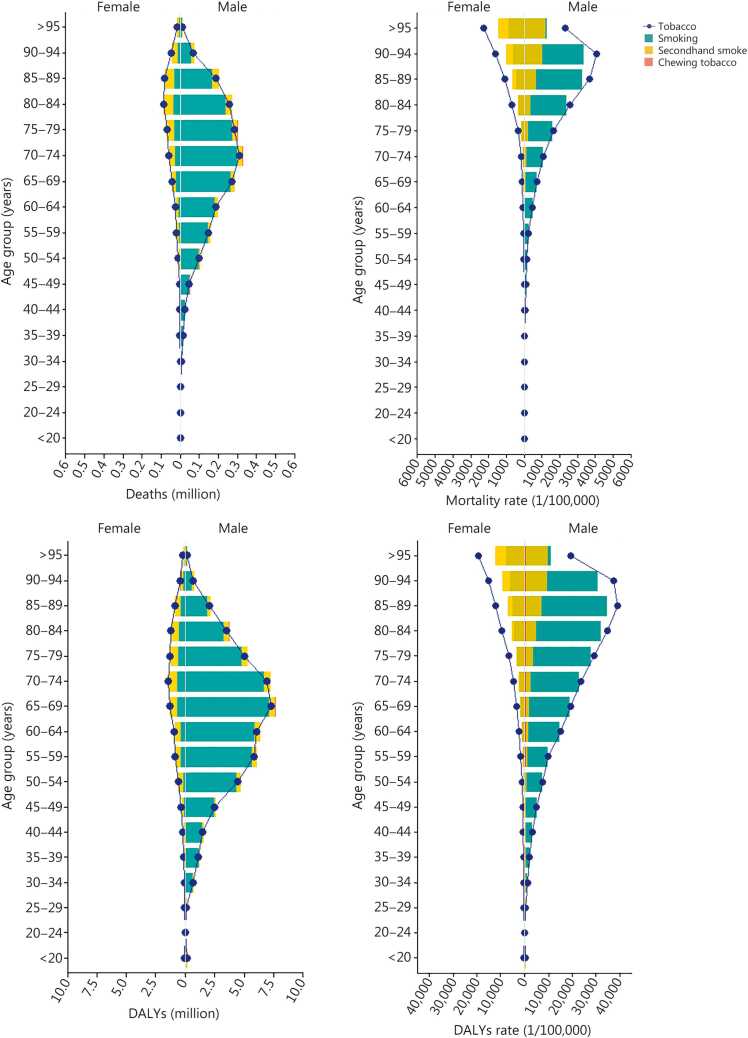
Age distributions of the numbers and rates of deaths and DALYs attributable to tobacco use, by sex and tobacco type in 2023. Stacked bars for deaths and DALYs. Overlaid bars for rates. DALY. Disability-adjusted life year.

The age-specific burden of tobacco use revealed pronounced disparities by sex and age. Across all age groups, the number of deaths attributable to tobacco was substantially higher in males than in females, with the disparity first widening and then narrowing with increasing age. Tobacco-attributable deaths were disproportionately concentrated in older populations, peaking among males aged 70−74 years and females aged 80−84 years (Fig. 2). A similar gradient was observed in mortality rates, which were consistently and markedly higher in males than in females across the age groups. The male mortality rates peaked notably in the 90–94-year age group, reflecting a heavy burden of tobacco-attributable fatalities in late life. In contrast, mortality counts among younger individuals (<40 years) remained very low (Fig. 2).

The age and sex patterns of DALYs closely mirrored those of deaths; however, the peak burden for DALYs occurred earlier, being concentrated in the 65–69-year age group. A notable finding was observed in the burden attributable to SHS. In contrast to the pattern for direct smoking, females experienced higher mortality and DALYs rate from SHS exposure than males did, with the disparity being most pronounced in individuals aged 70–74 years and older (Fig. 2).

### Disease spectrum attributable to tobacco use across the age groups

**Fig. 3:**
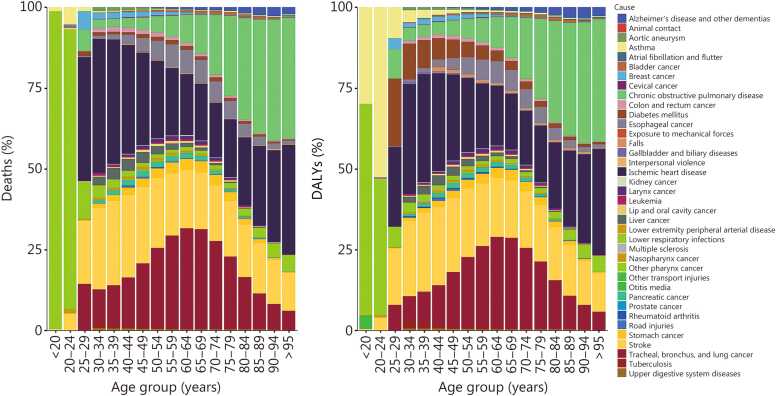
Burdens of tobacco-attributable deaths and DALYs across the lifespan in China in 2023. The results are presented by 5-year group. The left panel presents the proportion of age-specific deaths. The right panel presents the age-specific DALY proportion. Parkinson’s disease is not presented. DALY. Disability-adjusted life year.

Tobacco-attributable diseases show a clear and evolving pattern across the age groups, with deaths and DALYs following a broadly similar distribution (Fig. 3**; Additional file 1:** Table S6). In younger populations who were under 40 years of age, the tobacco-attributable burden was predominantly driven by lower respiratory infections and asthma. With advancing age, the disease spectrum shifted decisively toward chronic noncommunicable diseases and cancers (Fig. 3).

In people aged 25 years and older, tracheal, bronchus, and lung (TBL) cancer, ischemic heart disease (IHD), and stroke accounted for the largest shares of tobacco‑attributable deaths and DALYs, while lower respiratory infections accounted for a substantially smaller proportion (Fig. 3). In 2023, TBL cancer accounted for 22.18% of all tobacco‑attributable deaths and 21.08% of tobacco‑attributable DALYs. Together with COPD, these top 4 conditions constituted more than 80% of the total mortality burden, underscoring the overwhelming impact of tobacco on the cardiopulmonary system and airways in the aging population (**Additional file 1:** Table S6). A marked age-dependent increase was observed for COPD, whose proportional contribution to the total tobacco-attributable burden rose substantially with age, establishing it as a major driver of morbidity and mortality among older individuals (Fig. 3).

Furthermore, the rankings of the leading causes of death and DALYs were generally similar. However, some differences were observed. For example, diabetes mellitus ranked sixth and low back pain ranked seventh among the top 10 causes of DALYs, whereas they were less prominent in mortality rankings (Fig. 3**; Additional file 1:** Table S6).

### Geographical variation in the burden attributable to tobacco use

**Fig. 4:**
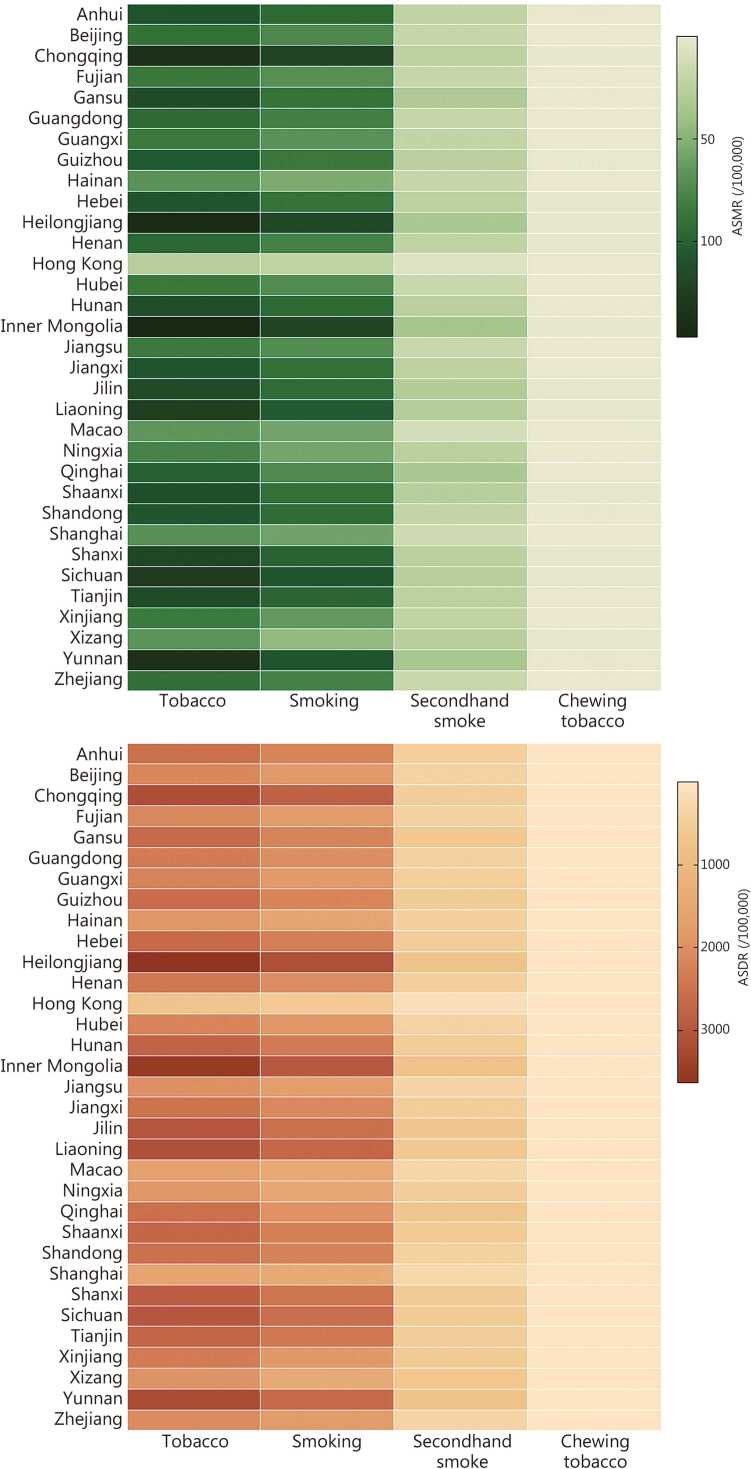
ASMR and ASDR across provinces by tobacco type in China in 2023. ASMR. Age-standardized mortality rate; ASDR. Age-standardized disability-adjusted life year rate.

Substantial geographical disparities in the ASRs of mortality and DALYs attributable to tobacco use were observed across China in 2023 (Fig. 4). The highest burden was concentrated in several northern and central provinces, with Heilongjiang, Inner Mongolia, and Chongqing emerging as key hotspots. In contrast, the burden was significantly lower in the economically developed coastal areas and some western provinces. Hong Kong, Shanghai, and Macao demonstrated notably lower ASMR and ASDR (Fig. 4). Additionally, ASMR were consistently higher among males than females across most provinces, with both sexes showing elevated ASMR and ASDR in northern regions and parts of southern China (**Additional file 1:** Figs. S1, S2).

While direct smoking remained the dominant source of tobacco-related mortality nationwide, provinces such as Inner Mongolia and Yunnan presented a disproportionately high share of their ASMRs attributable to SHS exposure (Fig. 4).

## Discussion

This analysis confirms that tobacco use imposes a multifaceted disease burden in China, characterized by a predominance of fatal outcomes. Smoking is the primary driver, disproportionately affecting males. Although ASRs have declined since 1990, absolute deaths and DALYs have risen, with distinct age and geographic patterns evident. These findings underscore the need for interventions tailored to these demographic and regional disparities.

### Paradoxical trend in burden

Our analysis revealed a critical divergence in the tobacco-attributable burden in China: while the absolute number of deaths and DALYs increased, their ASRs significantly declined. This pattern aligns with broader trends observed in other developing nations and has been attributed similarly to demographic forces, primarily population aging and growth [22], [23], [24]. A decomposition analysis for East Asia specifically identified population growth as the leading contributor to rising absolute counts, followed by changes in epidemiological rates [22], which means that a larger and older population expands the at-risk pool, so even a lower risk leads to a higher absolute burden. The decline in ASRs, underscored by a significant negative AAPC, is the expected result of reduced population-level risk. This success likely reflects the preliminary impact of China’s evolving tobacco control framework [25]. Additionally, it acknowledges the concurrent contributions of broader socioeconomic development, such as improvements in healthcare access and advances in the treatment of cardiovascular diseases and cancers [26], [27].

### Sex disparity

Males bore the majority of the smoking‑attributable disease burden in China. This pronounced sex disparity directly reflects differences in smoking prevalence [28], [29]. In many Western countries, such as France and Germany, smoking rates are relatively similar between sexes [28], [30]. In contrast, in several Asian countries, including China, India, and Indonesia, smoking is a predominantly male behavior, reflecting deeply entrenched sociocultural norms [28]. Consequently, whether measured by prevalence or the resulting disease burden, China and many other developing nations lag significantly behind high-income countries, with a tobacco epidemic that remains heavily skewed by sex.

In China, SHS accounts for a larger proportion of the total tobacco attributable burden in women than does direct smoking. This pattern reflects the substantial absolute burden of SHS exposure among women, which accounts for a disproportionately large share of their total tobacco attributable burden [31]. It thus reflects a critical gap in the implementation and enforcement of smoke-free measures and policies, especially in households and workplaces where exposure commonly occurs. This finding underscores the critical importance of protecting nonsmokers, particularly women, through stringent smoke-free legislation. Our analysis revealed a consistently minimal disease burden attributable to chewing tobacco in China. This indicates that, unlike the situation in some South Asian countries [24], [32], this form of tobacco use does not currently represent a major contributor to national burden.

### Differences in the age-specific disease spectrum

The disease spectrum attributable to tobacco use reveals a marked progression across the age groups. In younger populations, the burden is predominantly driven by conditions such as lower respiratory infections and asthma. With advancing age, the spectrum shifts decisively toward chronic noncommunicable diseases, where IHD, stroke, and TBL cancer emerge as the dominant causes of mortality and disability. This pattern is a direct manifestation of the cumulative and delayed pathogenesis of tobacco exposure [33]. Importantly, the health hazards of tobacco extend far beyond fatal cancers. Conditions such as diabetes mellitus and low back pain rank highly among the leading contributors to DALYs, underscoring that tobacco inflicts significant long-term disability and impairs quality of life through chronic pain and complex disease management [10], [34], [35]. While the International Agency for Research on Cancer (IARC) classifies tobacco as a Group 1 carcinogen, its health hazards are multifaceted, extending far beyond cancer [36], [37]. Given this broader spectrum of harm, a public health perspective is required that moves beyond a narrow focus on mortality. Such a perspective must fully account for the extensive harm tobacco imposes on population health and well-being across the age groups.

### Subregion differences

Mirroring global patterns of inequality, significant geographical disparities in the tobacco-attributable disease burden exist within China. A clear inverse correlation is observed between regional socioeconomic development and burden rates, with economically advanced coastal areas such as Hong Kong and Shanghai bearing a significantly lower burden than the national average [29], [38]. This divergence is not coincidental but is strongly suggestive of the decisive role of public health policy. Hong Kong and Shanghai, pioneers in tobacco control, have implemented some of the mainland’s most comprehensive and strictly enforced smoke-free legislation [39]. Their demonstrably lower ASMR and ASDR provide compelling evidence for the effectiveness of robust, well-enacted tobacco control policies in mitigating the population-level health impact [40].

These ASRs were disproportionately concentrated in several northern and central provinces, such as Heilongjiang, Inner Mongolia, and Chongqing. The elevated burden in these areas is likely multifactorial. First, a higher underlying prevalence of smoking may play a key role [41]. Second, contextual environmental factors such as poorer indoor ventilation during prolonged winter months, combined with gaps in the enforcement of smoke-free regulations, may lead to higher concentrations of SHS exposure in homes and workplaces [42], [43], [44], [45], [46]. Third, the potential synergistic effects between tobacco use and other prevalent risk factors, such as ambient air pollution, may exacerbate the risk of cardiovascular and respiratory diseases, contributing to the higher observed rates [47], [48]. To effectively reduce these geographical disparities, measurement design must move beyond a one-size-fits-all approach. Efforts must involve enhancing indoor air quality in northern provinces, intensifying cessation programs in high-prevalence areas, and strengthening local enforcement capacity in regions with poor compliance. These targeted efforts directly support the tobacco control targets outlined in the Healthy China 2030 initiative. By addressing the specific drivers of tobacco-attributable burdens in high-risk regions, this precision public health approach can accelerate progress toward the national goals of reducing premature mortality from noncommunicable diseases and improving overall population health equity.

### Synthesis and policy implications

Global tobacco control has achieved notable progress, as documented in recent WHO reports. However, this success is threatened by a counteroffensive from the tobacco industry, which aggressively markets new nicotine products to youth [49]. High-income countries have demonstrated that strong, evidence-based policies can effectively reduce smoking rates and related burdens [50]. Yet, the challenge remains complex in many countries, where demographic shifts, limited resources, and persistent industry tactics create distinct obstacles [51]. China exemplifies this complexity: despite declining ASRs, the absolute number of tobacco‑attributable deaths continues to rise, suggesting that current efforts are insufficient to counter population growth and aging.

Specifically, China’s burden profile dictates targeted imperatives. For men, who bear the majority of the direct smoking burden, robust and targeted cessation campaigns are essential; for women and children, strict enforcement of comprehensive smoke-free laws is crucial to reduce the significant SHS exposure driving their burden [52]. Efforts should focus on several priorities: 1) addressing the aging population by integrating tobacco control into chronic disease management; 2) tackling regional inequity by focusing resources on high-burden provinces; and 3) looking beyond mortality by prioritizing the reduction of tobacco-induced disability as a core metric for “Healthy China”. This evidence-based strategy is vital to transform preliminary progress into a decisive reversal of the tobacco epidemic’s health toll [53].

Several limitations warrant careful consideration when interpreting the results of this study. Firstly, a potential confounding factor is that some individuals may be exposed to both smoking and smokeless tobacco simultaneously. While the CRA framework employed in the GBD 2023 study has been refined to account for such overlapping risks to the greatest extent possible, it cannot guarantee complete statistical independence between these exposure pathways. This residual correlation may introduce a degree of uncertainty into our burden estimates. Secondly, our analysis relies substantially on self-reported data for tobacco use, which is susceptible to various reporting biases. These biases are not uniform and may systematically vary across different demographic strata, such as age, sex, geographical region, and socioeconomic status. Thirdly, a notable constraint of our study is that the analysis did not directly incorporate tobacco use prevalence data. This omission limits our ability to interpret the drivers of the observed burden trends directly. However, this gap is partially mitigated by the availability of numerous dedicated reports and studies focusing specifically on tobacco prevalence in China. These resources can be consulted to supplement and contextualize our findings.

## Conclusions

This study revealed that the tobacco-attributable burden is concentrated among males, older adults, and specific northern provinces, with a detailed disaggregation by direct smoking, SHS, and chewing tobacco. While ASRs have declined, indicating progress from national control measures, the absolute number of deaths and DALYs continues to rise. The findings underscore an urgent need for a more targeted and intensified policy response that addresses the distinct drivers of the burden in different subpopulations and regions. Ultimately, translating these granular findings into precise public health actions would be helpful for China to build on its preliminary success and achieve sustained progress against the tobacco epidemic.

## Abbreviations

AAPC, Average annual percentage change; ASDR, Age-standardized disability-adjusted life year rate; ASMR, Age-standardized mortality rate; ASRs, Age-standardized rates; BPRF, Burden of proof; COPD, Chronic obstructive pulmonary disease; CRA, Comparative risk assessment; DALYs, Disability-adjusted life years; GBD, Global Burden of Disease, Injuries, and Risk Factors Study; IHD, Ischemic heart disease; SHS, Secondhand smoke; TBL cancer, Tracheal, bronchus, and lung cancer; TMREL, Theoretical minimum risk exposure level; UI, Uncertainty interval; YLDs, Years lived with disability; YLLs, Years of life lost

## Ethics approval and consent to participate

Not applicable.

## Authors’ contributions

LLY and PY conceived this research. MGZ and PY provided overall guidance. LLY and FSY prepared the first draft and finalized the manuscript based on comments from all other authors. LLY, JLQ, LJW and PY contributed to the interpretation of the results, provided feedback on the initial drafts, and suggested improvements. All authors read and approved the final manuscript.

## Funding

Not applicable.

## Data Availability

The datasets analyzed during the current study are available on the Global Health Data Exchange GBD 2023 website (https://vizhub.healthdata.org/gbd-results/).

## Footnotes

Appendix A Supplementary data associated with this article can be found in the online version at doi:10.1016/j.mmr.2026.100041.
