# Supplementary material for: The disease burden attributable to tobacco use in China and its provinces from 1990−2023: an analysis from the Global Burden of Disease Study 2023
Source: Mil Med Res. 2026 May 23;13(1):100041. doi: 10.1016/j.mmr.2026.100041 (PMC13223522; doi:10.1016/j.mmr.2026.100041)
Supplement: Supplementary file 1 — Supplementary material [file mmc1.pdf]

Methods

Attributable burden estimates

Our analysis was conducted within the comparative risk assessment (CRA) framework of the Global Burden of Disease, Injuries, and Risk Factors Study (GBD) 2023, the methodological details of which have been extensively published elsewhere [1-3]. Consistent with the established definition of tobacco as a harmful risk factor with no safe level of exposure, the theoretical minimum risk exposure level (TMREL) was set at zero for all forms of tobacco use, indicating complete avoidance. The core metric for calculating the attributable burden is the population attributable fraction (PAF). For each tobacco-related outcome, the PAF was computed to represent the proportional reduction in disease burden that would occur if population exposure were reduced to the TMREL. The final burden attributable to tobacco, measured in deaths, disability-adjusted life years, years of life lost, and years lived with disability, was calculated by multiplying the PAFs for each outcome by their estimated disease burden.

Uncertainty analysis

Uncertainty was propagated throughout the entire estimation process to generate 95% UI for all final estimates, including exposure, PAFs, and attributable burden. This was achieved by 250 draws from the posterior distribution of each input parameter. The final mean estimate for each metric represents the mean value across these 250 draws [1]. The 95% UI was then calculated as the 2.5th and 97.5th percentiles of the ordered values from these draws [4].

Temporal trend analysis

Temporal trends in the tobacco-attributable disease burden were assessed using Joinpoint regression analysis. This methodology models temporal trends with a series of connected linear segments, allowing for the identification of significant inflection points, termed joinpoints, where the direction or rate of the trend changes. Joinpoint regression was selected over simple log-linear regression because it objectively determines the optimal number and location of these joinpoints via a grid search technique aimed at minimizing the sum of squared errors, thereby reducing subjectivity and providing a more accurate characterization of complex temporal patterns. Based on the 33-year study period from 1990 to 2023, we allowed a maximum of 6 joinpoints to adequately capture potential trend variations while maintaining model parsimony [5]. The statistical significance of identified trend changes was assessed via the Monte Carlo permutation method, implemented with the software's default setting of 4499 permutations to ensure robust inference.

Following the identification of the best-fitting Joinpoint model, the average annual percentage change (AAPC) was calculated as a weighted average of the annual percent changes from the model,

summarizing the average trend over the entire period [6]. A key advantage of the AAPC is that it remains a valid summary even when the Joinpoint model indicates fluctuations in the trend within the interval of interest. The 95% confidence interval (CI) for the AAPC was directly derived from the Joinpoint model, incorporating the uncertainty of the input data through its permutation testing framework. In our results, AAPC>0 indicates a statistically significant ascending trend, while AAPC<0 signifies a descending trend. All analyses were performed using Joinpoint software (version 5.4.0; National Cancer Institute, Rockville, MD, USA), with a P-value<0.05 considered statistically significant.

References

1. GBD 2023 Disease and Injury and Risk Factor Collaborators. Burden of 375 diseases and injuries, risk-attributable burden of 88 risk factors, and healthy life expectancy in 204 countries and territories, including 660 subnational locations, 1990-2023: a systematic analysis for the Global Burden of Disease Study 2023. Lancet. 2025;406(10513):1873-922. https://doi.org/10.1016/s0140-6736(25)01637-x
2. GBD 2015 Tobacco Collaborators. Smoking prevalence and attributable disease burden in 195 countries and territories, 1990-2015: a systematic analysis from the Global Burden of Disease Study 2015. Lancet. 2017;389(10082):1885-906. https://doi.org/10.1016/s0140-6736(17)30819-x
3. Murray CJ, Ezzati M, Lopez AD, Rodgers A, Vander Hoorn S. Comparative quantification of health risks conceptual framework and methodological issues. Popul Health Metr. 2003;1:1. https://doi.org/10.1186/1478-7954-1-1
4. Murray CJ, Lopez AD. Global mortality, disability, and the contribution of risk factors: Global Burden of Disease Study. Lancet. 1997;349(9063):1436-42. https://doi.org/10.1016/s0140-6736(96)07495-8
5. Number of joinpoints. https://surveillance.cancer.gov/help/joinpoint/setting-parameters/method-and-parameters-tab/number-of-joinpoints. Accessed 30 Oct 2025.
6. Average annual percent change (AAPC) and confidence interval. https://surveillance.cancer.gov/help/joinpoint/setting-parameters/method-and-parameters-tab/apc-aapc-tau-confidence-intervals/average-annual-percent-change-aapc. Accessed 30 Oct 2025.

Table S1 Data source of tobacco exposure and relative risk (*RR*) estimates

Data source of tobacco exposure	Data source of the *RR* of health outcomes associated with tobacco
Centers for Disease Control and Prevention (CDC) and World Health Organization (WHO). China-Global Youth Tobacco Survey 1999, Chongqing, Guangdong, Shandong and Tianjin. https://extranet.who.int/ncdsmicrodata/index.php/catalog/763/study-description. Accessed 15 May 2026.	Hu J, Johnson KC, Mao Y, Xu T, Lin Q, Wang C, *et al*. A case-control study of diet and lung cancer in northeast China. Int J Cancer. 1997;71(6):924-31.
Centers for Disease Control and Prevention (CDC) and World Health Organization (WHO). China-Global Youth Tobacco Survey 2005, Puyang, Shanghai, Tianjin, and Zhuhai. https://extranet.who.int/ncdsmicrodata/index.php/catalog/765/study-description. Accessed 15 May 2026.	Wong O, Harris F, Yiying W, Hua F. A hospital-based case-control study of acute myeloid leukemia in Shanghai: analysis of personal characteristics, lifestyle, and environmental risk factors by subtypes of the WHO classification. Regul Toxicol Pharmacol. 2009;55(3):340-52.
Chinese Center for Disease Control and Prevention (CCDC). China Chronic Disease and Risk Factor Surveillance 2004. https://ghdx.healthdata.org/record/china-chronic-disease-and-risk-factor-surveillance-2004. Accessed 15 May 2026.	Guo W, Blot WJ, Li JY, Taylor PR, Liu BQ, Wang W, *et al*. A nested case-control study of oesophageal and stomach cancers in the Linxian nutrition intervention trial. Int J Epidemiol. 1994;23(3):444-50.
Chinese Center for Disease Control and Prevention (CCDC). China Chronic Disease and Risk Factor Surveillance 2007. https://ghdx.healthdata.org/record/china-chronic-disease-and-risk-factor-surveillance-2007. Accessed 15 May 2026.	Guo LW, Lyu ZY, Meng QC, Zheng LY, Chen Q, Liu Y, *et al*. A risk prediction model for selecting high-risk populations for computed tomography lung cancer screening in China. Lung Cancer. 2022;163:27-34.
Chinese Center for Disease Control and Prevention. (CCDC). China Chronic Disease and Risk Factor Surveillance 2010. https://ghdx.healthdata.org/record/china-chronic-disease-and-risk-factor-surveillance-2010. Accessed 15 May 2026.	Chang ET, Liu Z, Hildesheim A, Liu Q, Cai Y, Zhang Z, *et al*. Active and Passive Smoking and Risk of Nasopharyngeal Carcinoma: A Population-Based Case-Control Study in Southern China. Am J Epidemiol. 2017;185(12):1272-80.

Data source of tobacco exposure	Data source of the *RR* of health outcomes associated with tobacco
Chinese Center for Disease Control and Prevention (CCDC). China Chronic Disease and Risk Factor Surveillance 2013. https://ghdx.healthdata.org/record/china-chronic-disease-and-risk-factor-surveillance-2013. Accessed 15 May 2026.	Zhu N, Yu C, Guo Y, Bian Z, Han Y, Yang L, *et al*, China Kadoorie Biobank Collaborative Group. Adherence to a healthy lifestyle and all-cause and cause-specific mortality in Chinese adults: a 10-year prospective study of 0.5 million people. Int J Behav Nutr Phys Act. 2019;16(1):98.
National Survey Research Center, Renmin University of China. China Comprehensive Social Survey 2011. http://css.cssn.cn/zgshzkzhdc/zlysj/lnsj/201706/t20170615_3551580.html. Accessed 15 May 2026.	Lv J, Yu C, Guo Y, Bian Z, Yang L, Chen Y, *et al*, China Kadoorie Biobank Collaborative Group. Adherence to a Healthy Lifestyle and the Risk of Type 2 Diabetes in Chinese Adults. Int J Epidemiol. 2017;46(5):1410-20.
CDC Foundation, Centers for Disease Control and Prevention (CDC), Chinese Center for Disease Control and Prevention (CCDC), Ministry of Health (China), World Health Organization (WHO). Global Adult Tobacco Survey (GATS) China 2010 Country Report. https://drupal.gtssacademy.org/wp-content/uploads/2024/11/GATS_China_2010_CountryReport_rev2012.pdf. Accessed 15 May 2026.	Lv J, Yu C, Guo Y, Bian Z, Yang L, Chen Y, *et al*. Adherence to Healthy Lifestyle and Cardiovascular Diseases in the Chinese Population. J Am Coll Cardiol. 2017;69(9):1116-25.
CDC Foundation, Centers for Disease Control and Prevention (CDC), Chinese Center for Disease Control and Prevention (CCDC), Ministry of Health (China), Research Triangle Institute, Inc. (RTI), World Health Organization (WHO). China Global Adult Tobacco Survey 2018. https://assets.tobaccofreekids.org/global/pdfs/en/GATS_China_2018_FactSheet.pdf. Accessed 15 May 2026.	Zeng J, Tang Y, Wu P, Fang X, Wang W, Fan Y, *et al*. Alcohol consumption, tobacco smoking, betel quid chewing and oral health associations with hypopharyngeal cancer among men in Central South China: a case-control study. Cancer Manag Res. 2019;11:6353-64.

Data source of tobacco exposure	Data source of the *RR* of health outcomes associated with tobacco
World Health Organization (WHO) and Centers for Disease Control and Prevention (CDC). Global School-Based Student Health Survey 2003, Beijing, Hangzhou, Wuhan, and Wurumqi. https://extranet.who.int/ncdsmicrodata/index.php/catalog/17. Accessed 15 May 2026.	Su WZ, Ohno Y, Tohnai I, Tamakoshi A, Wakai K, Yanbeet M, *et al*. Case-control study of oral cancer in Shenyang, Northeastern China. Int J Clin Oncol. 1998;3(13):13-18.
National Opinion Research Center, University of Chicago (NORC), Renmin University, Beijing, Peking Union Medical College, Beijing, University of North Carolina. China Health and Family Life Survey 1999–2000. https://august.csscr.washington.edu/~data/data8/publicdata/data/chfls/CHFLSEnglish1pdf.pdf. Accessed 15 May 2026.	He Y, Jiang B, Li LS, Li LS, Sun DL, Wu L, *et al*. Changes in smoking behavior and subsequent mortality risk during a 35-year follow-up of a cohort in Xi'an, China. Am J Epidemiol. 2014;179(9):1060-70.
Zhang, B, Zhai, F, Du, S, Popkin, B. (2014). The China Health and Nutrition Survey, 1989-2011. Obesity Reviews, 15 (Suppl.1), 2–7.	Ji BT, Chow WH, Dai Q, McLaughlin JK, Benichou J, Hatch MC, *et al*. Cigarette smoking and alcohol consumption and the risk of pancreatic cancer: a case-control study in Shanghai, China. Cancer Causes Control. 1995;6(4):369-76.
Carolina Population Center, University of North Carolina at Chapel Hill, Chinese Center for Disease Control and Prevention (CCDC). China Health and Nutrition Survey 1991. https://chns.cpc.unc.edu/. Accessed 15 May 2026.	Kelly TN, Gu D, Chen J, Huang JF, Chen JC, Duan X, *et al*. Cigarette smoking and risk of stroke in the Chinese adult population. Stroke. 2008;39(6):1688-93.
Carolina Population Center, University of North Carolina at Chapel Hill, Chinese Center for Disease Control and Prevention (CCDC). China Health and Nutrition Survey 1993. https://chns.cpc.unc.edu/. Accessed 15 May 2026.	Hou X, Qiu J, Chen P, Lu J, Ma X, Lu J, *et al*. Cigarette Smoking Is Associated with a Lower Prevalence of Newly Diagnosed Diabetes Screened by OGTT than Non-Smoking in Chinese Men with Normal Weight. PLoS One. 2016;11(3):e0149234.

Data source of tobacco exposure	Data source of the *RR* of health outcomes associated with tobacco
Carolina Population Center, University of North Carolina at Chapel Hill, Chinese Center for Disease Control and Prevention (CCDC). China Health and Nutrition Survey 1997. https://chns.cpc.unc.edu/. Accessed 15 May 2026.	Jin ZY, Wallar G, Zhou JY, Yang J, Han RQ, Wang PH, *et al*. Consumption of garlic and its interactions with tobacco smoking and alcohol drinking on esophageal cancer in a Chinese population. Eur J Cancer Prev. 2019;28(4):278-86.
Carolina Population Center, University of North Carolina at Chapel Hill, Chinese Center for Disease Control and Prevention (CCDC). China Health and Nutrition Survey 2000. https://chns.cpc.unc.edu/. Accessed 15 May 2026.	Zheng W, Blot WJ, Shu XO, Gao YT, Ji BT, Ziegler RG, *et al*. Diet and other risk factors for laryngeal cancer in Shanghai, China. Am J Epidemiol. 1992;136(2):178-91.
Carolina Population Center, University of North Carolina at Chapel Hill, Chinese Center for Disease Control and Prevention (CCDC). China Health and Nutrition Survey 2004. https://chns.cpc.unc.edu/. Accessed 15 May 2026.	Chen ZM, Xu Z, Collins R, Li WX, Peto R. Early health effects of the emerging tobacco epidemic in China. A 16-year prospective study. JAMA. 1997;278(18):1500-4.
Carolina Population Center, University of North Carolina at Chapel Hill, Chinese Center for Disease Control and Prevention (CCDC). China Health and Nutrition Survey 2006. https://chns.cpc.unc.edu/. Accessed 15 May 2026.	Liu BQ, Peto R, Chen ZM, Boreham J, Wu YP, Li JY, *et al*. Emerging tobacco hazards in China: 1. Retrospective proportional mortality study of one million deaths. BMJ. 1998;317(7170):1411-22.
China Center for Economic Research, Peking University. China Health and Retirement Longitudinal Study 2008. https://charls.charlsdata.com/pages/Data/2008-charls-pilot/zh-cn.html. Accessed 15 May 2026.	Yuan JM, Ross RK, Wang XL, Gao YT, Henderson BE, Yu MC. Morbidity and mortality in relation to cigarette smoking in Shanghai, China. A prospective male cohort study. JAMA. 1996;275(21):1646-50.
China Center for Economic Research, Peking University. China Health and Retirement Longitudinal Study 2011−2012. https://charls.charlsdata.com/pages/Data/2011-charls-wave1/zh-cn.html. Accessed 15 May 2026.	Lam TH, Ho SY, Hedley AJ, Mak KH, Peto R. Mortality and smoking in Hong Kong: case-control study of all adult deaths in 1998. BMJ. 2001;323(7309):361.

Data source of tobacco exposure	Data source of the *RR* of health outcomes associated with tobacco
China Center for Economic Research, Peking University. China Health and Retirement Longitudinal Study Pilot Resurvey 2012. https://charls.charlsdata.com/pages/Data/2012-charls-pilot-wave2/zh-cn.html. Accessed 15 May 2026.	Ji X, Zhang W, Xie C, Wang B, Zhang G, Zhou F. Nasopharyngeal carcinoma risk by histologic type in central China: impact of smoking, alcohol and family history. Int J Cancer. 2011;129(3):724-32.
China Center for Economic Research, Peking University. China Health and Retirement Longitudinal Study, Wave 2 2013. https://charls.charlsdata.com/pages/Data/2013-charls-wave2/zh-cn.html. Accessed 15 May 2026.	Yuan JM, Wang XL, Xiang YB, Gao YT, Ross RK, Yu MC. Non-dietary risk factors for nasopharyngeal carcinoma in Shanghai, China. Int J Cancer. 2000;85(3):364-9.
National Bureau of Statistics of China. China Health Statistics Yearbook 2006. https://www.stats.gov.cn/sj/ndsj/2006/indexeh.htm. Accessed 15 May 2026.	Zhang T, Yang X, Yin X, Yuan Z, Chen H, Jin L, *et al*. Poor oral hygiene behavior is associated with an increased risk of gastric cancer: A population-based case-control study in China. J Periodontol. 2022;93(7):988-1002
Riskin, Carl, Zhao Renwei, and Li Shi. Chinese Household Income Project, 1995. https://www.icpsr.umich.edu/web/DSDR/studies/3012. Accessed 15 May 2026.	Tse LA, Wang F, Wong MC, Au JS, Yu IT. Risk assessment and prediction for lung cancer among Hong Kong Chinese men. BMC Cancer. 2022;22(1):585.
Center for Healthy Aging and Family Studies, Peking University, China Mainland Information Group, China Research Center on Aging, Duke University, Max Planck Institute for Demographic Research. China Longitudinal Healthy Longevity Survey 1998−2009. https://opendata.pku.edu.cn/dataset.xhtml?persistentId=doi:10.18170/DVN/WBO7LK&version=2.0. Accessed 15 May 2026.	Yu Y, Hu J, Wang PP, Zou Y, Qi Y, Zhao P, *et al*. Risk factors for bladder cancer: a case-control study in northeast China. Eur J Cancer Prev. 1997;6(4):363-9.
Ministry of Health (China). China National Health Services Survey 2003. https://www.nhc.gov.cn/mohwsbwstjxxzx/s8211/201009/bcb37f634b4e497ea6f49120387607a2/files/1740020360672_22754.pdf. Accessed 15 May 2026.	Gao YT, McLaughlin JK, Blot WJ, Ji BT, Benichou J, Dai Q, *et al*. Risk factors for esophageal cancer in Shanghai, China. I. Role of cigarette smoking and alcohol drinking. Int J Cancer. 1994;58(2):192-6.

**Data source of tobacco exposure**	**Data source of the *RR* of health outcomes associated with tobacco**
Ministry of Health (China). China National Health Services Survey 2008. https://www.nhc.gov.cn/mohwsbwstjxxzx/s8211/201009/768f45b7ef2c46e 7a2b2a603b0f4ec0e/files/1740020418481_84545.pdf. Accessed 15 May 2026.	Wang JY, Liu SB, Chen SY, Dobson A. Risk factors for peptic ulcer in Shanghai. Int J Epidemiol. 1996;25(3):638-43.
National Bureau of Statistics of China, Minnesota Population Center. China National Population Census 1982. https://scalar.usc.edu/works/chinese-studies-reference-collection/media/HB3654.A3%20C54X%20Zhongguo% 201982%20nian%20renkou%20pucha%20ziliao_OCR.pdf. Accessed 15 May 2026.	Wang JG, Staessen JA, Fagard R, Gong L, Liu L, Systolic Hypertension in China (Syst-China) Trial Collaborative Group. Risks of smoking in treated and untreated older Chinese patients with isolated systolic hypertension. J Hypertens. 2001;19(2):187-92.
National Bureau of Statistics of China and Minnesota Population Center. China National Population Census 1990. https://ghdx.healthdata.org/record/china-national-population-census-1990-ipums. Accessed 15 May 2026.	Fu JY, Gao J, Zhang ZY, Zheng JW, Zhong LP, Luo JF, *et al*. Role of cigarette filter on the risk of oral cancer: a case-control study in a Chinese population. Oral Dis. 2013;19(1):80-4.
Chinese Academy of Preventive Medicine, Chinese Association on Smoking and Health, Johns Hopkins University, Ministry of Health (China). China National Prevalence Survey on Smoking 1996. https://ghdx.healthdata.org/record/china-national-prevalence-survey-smoking-1996. Accessed 15 May 2026.	Stern MC, Van Den Berg D, Yuan JM, Conti DV, Gago-Dominguez M, Pike MC, *et al*. Sequence variant on 3q28 and urinary bladder cancer risk: findings from the Los Angeles-Shanghai bladder case-control study. Cancer Epidemiol Biomarkers Prev. 2009;18(11):3057-61.
China Population Information and Research Center. China Population and Housing Census 1990 - China Archive. https://ghdx.healthdata.org/record/china-population-and-housing-census-1990-china-archive. Accessed 15 May 2026.	Li L, Li SY, Zhong X, Ren J, Tian X, Tuerxun M, *et al*. SERPINE2 rs16865421 polymorphism is associated with a lower risk of chronic obstructive pulmonary disease in the Uygur population: A case-control study. J Gene Med. 2019;21(9):e3106.

**Data source of tobacco exposure**	**Data source of the *RR* of health outcomes associated with tobacco**
World Health Organization (WHO). China World Health Survey 2002. https://ghdx.healthdata.org/record/china-world-health-survey-2002. Accessed 15 May 2026.	Lin JH, Jiang CQ, Ho SY, Zhang WS, Mai ZM, Xu L, *et al*. Smoking and Nasopharyngeal Carcinoma Mortality: a Cohort Study of 101,823 Adults in Guangzhou, China. BMC Cancer. 2015;15:906.
Ministry of Health (China), National Center for Chronic and Noncommunicable Disease Control and Prevention, Chinese Center for Disease Control and Prevention (CCDC), World Health Organization (WHO). China WHO Study on Global Ageing and Adult Health 2007–2010. https://apps.who.int/healthinfo/systems/surveydata/index.php/catalog/13#study_desc1684335103457. Accessed 15 May 2026.	Xu L, Schooling CM, Chan WM, Lee SY, Leung GM, Lam TH. Smoking and hemorrhagic stroke mortality in a prospective cohort study of older Chinese. Stroke. 2013;44(8):2144-9.
Gallup. China World Poll 2005–2006. https://ghdx.healthdata.org/record/china-world-poll-2005-2006. Accessed 15 May 2026.	Liu ZY, He XZ, Chapman RS. Smoking and other risk factors for lung cancer in Xuanwei, China. Int J Epidemiol. 1991;20(1):26-31.
Gallup. China World Poll 2009. https://ghdx.healthdata.org/record/china-world-poll-2009. Accessed 15 May 2026.	Leung CC, Li T, Lam TH, Yew WW, Law WS, Tam CM, *et al*. Smoking and tuberculosis among the elderly in Hong Kong. Am J Respir Crit Care Med. 2004;170(9):1027-33.
Gallup. China World Poll 2011. https://ghdx.healthdata.org/record/china-world-poll-2011. Accessed 15 May 2026.	Lam TH, Li ZB, Ho SY, Chan WM, Ho KS, Tham MK, *et al*. Smoking, quitting and mortality in an elderly cohort of 56,000 Hong Kong Chinese. Tob Control. 2007;16(3):182-9.
Gallup. China World Poll 2012. https://ghdx.healthdata.org/record/china-world-poll-2012. Accessed 15 May 2026.	Lei YX, Cai WC, Chen YZ, Du YX. Some lifestyle factors in human lung cancer: a case-control study of 792 lung cancer cases. Lung Cancer. 1996;14 Suppl 1:S121-36.

**Data source of tobacco exposure**	**Data source of the *RR* of health outcomes associated with tobacco**
Institute of Social Science Survey, University of Peking. Chinese Family Panel Studies Baseline 2010–2011. https://www.isss.pku.edu.cn/cfps/sjzx/gksj/index.htm. Accessed 15 May 2026.	Ji BT, Chow WH, Yang G, McLaughlin JK, Gao RN, Zheng W, *et al*. The influence of cigarette smoking, alcohol, and green tea consumption on the risk of carcinoma of the cardia and distal stomach in Shanghai, China. Cancer. 1996;77(12):2449-57.
Centers for Disease Control and Prevention (CDC), World Health Organization (WHO). Hong Kong Global Youth Tobacco Survey 2009. https://ghdx.healthdata.org/record/hong-kong-global-youth-tobacco-survey-2009. Accessed 15 May 2026.	Jin K, Wu M, Zhou JY, Yang J, Han RQ, Jin ZY, *et al*. Tobacco Smoking Modifies the Association between Hormonal Factors and Lung Cancer Occurrence among Post-Menopausal Chinese Women. Transl Oncol. 2019;12(6):819-27.
Gallup. Hong Kong World Poll 2005–2006. https://ghdx.healthdata.org/record/hong-kong-world-poll-2005-2006. Accessed 15 May 2026.	-
Gallup. Hong Kong World Poll 2012. https://ghdx.healthdata.org/record/hong-kong-world-poll-2012. Accessed 15 May 2026.	-
Centers for Disease Control and Prevention (CDC) and World Health Organization (WHO). Macao Global Youth Tobacco Survey 2001. https://extranet.who.int/ncdsmicrodata/index.php/catalog/909/study-description. Accessed 15 May 2026.	-
Centers for Disease Control and Prevention (CDC) and World Health Organization (WHO). Macao Special Administrative Region of China Global Youth Tobacco Survey 2005. https://extranet.who.int/ncdsmicrodata/index.php/catalog/908/study-description. Accessed 15 May 2026.	-

Data source of tobacco exposure	Data source of the *RR* of health outcomes associated with tobacco
Centers for Disease Control and Prevention (CDC), World Health Organization (WHO). Macao Global Youth Tobacco Survey 2010. https://extranet.who.int/ncdsmicrodata/index.php/catalog/702. Accessed 15 May 2026.	-
Centers for Disease Control and Prevention (CDC), Health Bureau (Macao), World Health Organization (WHO). Macao Global Youth Tobacco Survey 2015. https://cdn.who.int/media/docs/default-source/ncds/ncd-surveillance/data-reporting/macao/macao---sar-gyts-2015-factsheet-(ages-13-15)tag508.pdf?sfvrsn=62c13716_1. Accessed 15 May 2026.	-
Weng XZ, Hong ZG, Chen DY. Smoking prevalence in the Chinese aged 15 and above. Chin Med J (Engl). 1987; 100(11): 886-92	-
World Health Organization (WHO). WHO Report on the Global Tobacco Epidemic 2019 : offer help to quit tobacco use. https://digitallibrary.un.org/record/4058519?v=pdf. Accessed 15 May 2026.	-

Table S2 Level 3 risk-outcome attributable to tobacco use

Cause name	Status
Smoking	
Latent tuberculosis infection	Used in GBD 2021 and GBD 2023
Drug-susceptible tuberculosis	Used in GBD 2021 and GBD 2023
Multidrug-resistant tuberculosis without extensive drug resistance	Used in GBD 2021 and GBD 2023
Extensively drug-resistant tuberculosis	Used in GBD 2021 and GBD 2023
Lower respiratory infections	Used in GBD 2021 and GBD 2023
Lip and oral cavity cancer	Used in GBD 2021 and GBD 2023
Nasopharynx cancer	Used in GBD 2021 and GBD 2023
Other pharynx cancer	Used in GBD 2021 and GBD 2023
Oesophageal cancer	Used in GBD 2021 and GBD 2023
Stomach cancer	Used in GBD 2021 and GBD 2023
Colon and rectum cancer	Used in GBD 2021 and GBD 2023
Liver cancer due to hepatitis B	Used in GBD 2021 and GBD 2023
Liver cancer due to hepatitis C	Used in GBD 2021 and GBD 2023
Liver cancer due to alcohol use	Used in GBD 2021 and GBD 2023
Liver cancer due to NASH	Used in GBD 2021 and GBD 2023
Liver cancer due to other causes	Used in GBD 2021 and GBD 2023
Pancreatic cancer	Used in GBD 2021 and GBD 2023
Larynx cancer	Used in GBD 2021 and GBD 2023
Tracheal, bronchus, and lung cancer	Used in GBD 2021 and GBD 2023
Breast cancer	Used in GBD 2021 and GBD 2023
Cervical cancer	Used in GBD 2021 and GBD 2023
Prostate cancer	Used in GBD 2021 and GBD 2023
Kidney cancer	Used in GBD 2021 and GBD 2023
Bladder cancer	Used in GBD 2021 and GBD 2023
Acute lymphoid leukaemia	Used in GBD 2021 and GBD 2023
Chronic lymphoid leukaemia	Used in GBD 2021 and GBD 2023
Acute myeloid leukaemia	Used in GBD 2021 and GBD 2023
Chronic myeloid leukaemia	Used in GBD 2021 and GBD 2023
Other leukaemia	Used in GBD 2021 and GBD 2023
Ischaemic heart disease	Used in GBD 2021 and GBD 2023
Ischaemic stroke	Used in GBD 2021 and GBD 2023
Intracerebral haemorrhage	Used in GBD 2021 and GBD 2023
Subarachnoid haemorrhage	Used in GBD 2021 and GBD 2023

Cause name	Status
Atrial fibrillation and flutter	Used in GBD 2021 and GBD 2023
Aortic aneurysm	Used in GBD 2021 and GBD 2023
Lower extremity peripheral arterial disease	Used in GBD 2021 and GBD 2023
Chronic obstructive pulmonary disease	Used in GBD 2021 and GBD 2023
Asthma	Used in GBD 2021 and GBD 2023
Peptic ulcer disease	Used in GBD 2021 and GBD 2023
Gallbladder and biliary diseases	Used in GBD 2021 and GBD 2023
Alzheimer’s disease and other dementias	Used in GBD 2021 and GBD 2023
Parkinson’s disease	Used in GBD 2021 and GBD 2023
Multiple sclerosis	Used in GBD 2021 and GBD 2023
Diabetes mellitus type 2	Used in GBD 2021 and GBD 2023
Cataract	Used in GBD 2021 and GBD 2023
Age-related macular degeneration	Used in GBD 2021 and GBD 2023
Rheumatoid arthritis	Used in GBD 2021 and GBD 2023
Low back pain	Used in GBD 2021 and GBD 2023
Pedestrian road injuries	Used in GBD 2021 and GBD 2023
Cyclist road injuries	Used in GBD 2021 and GBD 2023
Motorcyclist road injuries	Used in GBD 2021 and GBD 2023
Motor vehicle road injuries	Used in GBD 2021 and GBD 2023
Other road injuries	Used in GBD 2021 and GBD 2023
Other transport injuries	Used in GBD 2021 and GBD 2023
Falls	Used in GBD 2021 and GBD 2023
Other exposure to mechanical forces	Used in GBD 2021 and GBD 2023
Non-venomous animal contact	Used in GBD 2021 and GBD 2023
Physical violence by other means	Used in GBD 2021 and GBD 2023
Secondhand smoke	
Lower respiratory infections	Used in GBD 2021 and GBD 2023
Otitis media	Used in GBD 2021 and GBD 2023
Tracheal, bronchus, and lung cancer	Used in GBD 2021 and GBD 2023
Breast cancer	Used in GBD 2021 and GBD 2023
Ischaemic heart disease	Used in GBD 2021 and GBD 2023
Ischaemic stroke	Used in GBD 2021 and GBD 2023
Intracerebral haemorrhage	Used in GBD 2021 and GBD 2023
Subarachnoid haemorrhage	Used in GBD 2021 and GBD 2023
Chronic obstructive pulmonary disease	Used in GBD 2021 and GBD 2023

Cause name	Status
Asthma	Added in GBD 2023
Diabetes mellitus type 2	Used in GBD 2021 and GBD 2023
Chewing tobacco	
Lip and oral cavity cancer	Used in GBD 2021 and GBD 2023
Nasopharynx cancer	Added in GBD 2023
Other pharynx cancer	Added in GBD 2023
Oesophageal cancer	Used in GBD 2021 and GBD 2023
Larynx cancer	Added in GBD 2023
Ischaemic stroke	Added in GBD 2023
Intracerebral haemorrhage	Added in GBD 2023
Subarachnoid haemorrhage	Added in GBD 2023

NASH. Nonalcoholic steatohepatitis; GBD. Global Burden of Diseases, Injuries, and Risk Factors Study

Table S3 List of International Classification of Diseases (ICD) codes mapped to causes attributable to tobacco use at Level 3 of the GBD risk taxonomy

Cause	ICD-10	ICD-9
Alzheimer’s disease and other dementias	F00−F02.0, F02.8−F03.9, G30−G31.1, G31.8−G31.9	290−290.9, 294.1−294.9, 331−331.2
Animal contact	W52.0−W62.9, W64−W64.9, X20−X29.9	E905−E906
Aortic aneurysm	I71−I71.9	441−441.9
Asthma	J45−J46.9	493−493.9
Atrial fibrillation and flutter	I48−I48.9	427.3
Bladder cancer	C67−C67.9, D09.0, D30.3, D41.4−D41.8, D49.4	188−188.9, 223.3, 233.7, 236.7, 239.4
Breast cancer	C50−C50.9, D05−D05.9, D24−D24.9, D48.6, D49.3	174−175.9, 217−217.8, 233.0, 238.3, 239.3, 610−610.9
Cervical cancer	C53−C53.9, D06−D06.9, D26.0	180−180.9, 219.0, 233.1, 622.1−622.2, 622.7
Chronic obstructive pulmonary disease	J41−J44.9	491−492.9, 496−499
Colon and rectum cancer	C18−C21.9, D01.0−D01.3, D12−D12.9, D37.3−D37.5	153−154.9, 209.1, 209.5, 211.3−211.4, 230.3−230.6, 569.0
Diabetes mellitus	E10−E10.1, E10.3−E11.1, E11.3−E11.9, P70.2	775.1
Esophageal cancer	C15−C15.9, D00.1, D13.0	150−150.9, 211.0, 230.1
Exposure to mechanical forces	W20−W38.9, W40−W43.9, W45.0−W45.2, W46−W46.2, W49−W52	E916−E922
Falls	W00−W19.9	E880−E886, E888
Gallbladder and biliary diseases	K80−K83.9	574−576.9

Cause	ICD-10	ICD-9
Interpersonal violence	X85−Y08.9, Y87.1	E960−E969
Ischemic heart disease	I20−I25.9	410−414.9
Kidney cancer	C64−C65.9, D30.0−D30.1, D41.0−D41.1	189.0−189.1, 189.5−189.6, 223.0−223.1
Larynx cancer	C32−C32.9, D02.0, D14.1, D38.0	161−161.9, 212.1, 231.0, 235.6
Leukemia	C91−C91.0, C91.2−C91.3, C91.6, C92−C92.6, C93−C93.1, C93.3, C93.8, C94−C94.5, C94.7−C95.9	204−204.0, 204.2, 205−205.3, 206−206.1, 207−208.9
Lip and oral cavity cancer	C00−C08.9, D10.0−D10.5, D11−D11.9	140−145.9, 210.0−210.6, 235.0
Liver cancer	C22−C22.8, D13.4	155−155.1, 155.3−155.9, 211.5
Lower extremity peripheral arterial disease	I70.2−I70.8, I73−I73.9	440.2, 440.4, 443.0−443.9
Lower respiratory infections	A48.1, A70, B34.2, B97.2, B97.4−B97.6, J09−J15.8, J16−J16.9, J20−J21.9, J91.0, P23.0−P23.4, U04−U04.9	079.6, 466−469, 470.0, 480−482.8, 483.0−483.9, 484.1−484.2, 484.6−484.7, 487−489
Multiple sclerosis	G35−G35.9	340−340.9
Nasopharynx cancer	C11−C11.9, D10.6	147−147.9, 210.7−210.9
Other pharynx cancer	C09−C10.9, C12−C13.9, D10.7	146−146.9, 148−148.9
Other transport injuries	V00−V00.8, V05−V05.9, V81−V81.9, V83−V86.9, V88.2−V88.3, V90−V98.8	E800−E807, E830−E838, E840−E849
Otitis media	H70−H70.9	381−383.9
Pancreatic cancer	C25−C25.9, D13.6−D13.7	157−157.9, 211.6−211.7

Cause	ICD-10	ICD-9
Prostate cancer	C61−C61.9, D07.5, D29.1, D40.0	185−185.9, 222.2, 236.5
Rheumatoid arthritis	M05−M06.9, M08.0−M08.8	714−714.3, 714.8−714.9
Road injuries	V01−V04.99, V06−V80.929, V82−V82.9, V87.2−V87.3	E800.3, E801.3, E802.3, E803.3, E804.3, E805.3, E806.3, E807.3, E810.0−E810.6, E811.0−E811.7, E812.0−E812.7, E813.0−E813.7, E814.0−E814.7, E815.0−E815.7, E816.0−E816.7, E817.0−E817.7, E818.0−E818.7, E819.0−E819.7, E820.0−E820.6, E821.0−E821.6, E822.0−E822.7, E823.0−E823.7, E824.0−E824.7, E825.0−E825.7, E826.0−E826.1, E826.3−E826.4, E827.0, E827.3−E827.4, E828.0, E828.4, E829.0−E829.4, V03, V07.8−V07.9, V13, V13.8, V13.9, V15.2, V15.3, V15.9, V19, V42, V42.8, V42.9−V43, V47−V47.1
Stomach cancer	C16−C16.9, D00.2, D13.1, D37.1	151−151.9, 211.1, 230.2
Stroke	G45−G46.8, I60−I63.9, I65−I66.9, I67.0−I67.3, I67.5−I67.6, I68.1−I68.2, I69.0−I69.3	430−435.9, 437.0−437.2, 437.5−437.8
Tracheal, bronchus, and lung cancer	C33−C34.9, D02.1−D02.3, D14.2−D14.3, D38.1	162−162.9, 212.2−212.3, 231.1−231.2, 235.7
Tuberculosis	A10−A14, A15−A19.9, B90−B90.9, K67.3, K93.0, M49.0, N74.1, P37.0, U84.3	010−019.9, 137−137.9, 138.0−138.9, 320.4, 730.4−730.6
Upper digestive system diseases	K25−K29.9	531−535.9

ICD. International Classification of Diseases

Table S4 Percentage changes in numbers and age-standardized rates of tobacco-attributable burden, by sex and tobacco use type, 1990–2023

Tobacco type	Deaths		DALYs		YLDs		YLLs	
	**Number (95% UI)**	**ASR (/100,000, 95% UI)**	**Number (95% UI)**	**ASR (/100,000, 95% UI)**	**Number (95% UI)**	**ASR (/100,000, 95% UI)**	**Number (95% UI)**	**ASR (/100,000, 95% UI)**
Tobacco								
Both	0.34 (0.11–0.61)	−0.55 (−0.63 to −0.45)	0.09 (−0.08 to 0.27)	−0.58 (−0.64 to −0.50)	0.90 (0.65−1.20)	−0.18 (−0.29 to −0.05)	0.02 (−0.15 to 0.20)	−0.61 (−0.67 to −0.53)
Male	0.38 (0.14–0.69)	−0.54 (−0.63 to −0.44)	0.16 (−0.04 to 0.37)	−0.55 (−0.63 to −0.46)	0.97 (0.75−1.21)	−0.12 (−0.22 to −0.02)	0.09 (−0.11 to 0.31)	−0.58 (−0.66 to −0.49)
Female	0.17 (−0.11 to 0.52)	−0.64 (−0.72 to −0.54)	−0.16 (−0.33 to 0.08)	−0.68 (−0.75 to −0.60)	0.68 (0.29−1.20)	−0.32 (−0.48 to −0.10)	−0.24 (−0.40 to −0.02)	−0.72 (−0.78 to −0.64)
Smoking								
Both	0.37 (0.11–0.68)	−0.54 (−0.64 to −0.43)	0.22 (0.01–0.47)	−0.53 (−0.62 to −0.44)	0.89 (0.59−1.22)	−0.20 (−0.32 to −0.05)	0.16 (−0.06 to 0.41)	−0.56 (−0.65 to −0.47)
Male	0.40 (0.15–0.72)	−0.54 (−0.63 to −0.42)	0.26 (0.05–0.5)	−0.52 (−0.6 to −0.42)	0.97 (0.74−1.21)	−0.13 (−0.24 to −0.03)	0.19 (−0.03 to 0.45)	−0.55 (−0.64 to −0.45)
Female	0.11 (−0.31 to 0.83)	−0.64 (−0.88 to 0.14)	−0.03 (−0.40 to 0.61)	−0.67 (−0.80 to −0.46)	0.44 (−0.08 to 1.44)	−0.48 (−0.67 to −0.1)	−0.09 (−0.44 to 0.52)	−0.70 (−0.81 to −0.50)
Secondhand smoke								
Both	0.24 (−0.04 to 0.56)	−0.6 (−0.69 to −0.5)	−0.28 (−0.43 to −0.11)	−0.70 (−0.76 to −0.63)	0.98 (0.64−1.37)	−0.08 (−0.23 to 0.08)	−0.35 (−0.49 to −0.18)	−0.73 (−0.78 to −0.66)
Male	0.27 (−0.09 to 0.70)	−0.59 (−0.70 to −0.44)	−0.34 (−0.52 to −0.13)	−0.70 (−0.78 to −0.6)	1.07 (0.62−1.67)	0.02 (−0.20 to 0.28)	−0.39 (−0.56 to −0.19)	−0.73 (−0.80 to −0.64)
Female	0.22 (−0.03 to 0.57)	−0.67 (−0.80 to −0.46)	−0.23 (−0.38 to −0.03)	−0.69 (−0.75 to −0.62)	0.93 (0.65−1.22)	−0.13 (−0.25 to −0.02)	−0.31 (−0.45 to −0.12)	−0.73 (−0.78 to −0.66)

Tobacco type	Deaths		DALYs		YLDs		YLLs	
	**Number (95% UI)**	**ASR (/100,000, 95% UI)**	**Number (95% UI)**	**ASR (/100,000, 95% UI)**	**Number (95% UI)**	**ASR (/100,000, 95% UI)**	**Number (95% UI)**	**ASR (/100,000, 95% UI)**
Chewing tobacco								
Both	0.42 (−0.50 to 3.33)	−0.51 (−0.82 to 0.52)	0.21 (−0.57 to 2.76)	−0.51 (−0.83 to 0.53)	1.85 (0−8.02)	0.31 (−0.54 to 3.07)	0.15 (−0.59 to 2.56)	−0.54 (−0.84 to 0.44)
Male	0.53 (−0.46 to 3.63)	−0.45 (−0.80 to 0.68)	0.31 (−0.53 to 3.02)	−0.45 (−0.80 to 0.68)	1.98 (0.05−8.36)	0.38 (−0.51 to 3.28)	0.25 (−0.55 to 2.84)	−0.48 (−0.81 to 0.60)
Female	0.12 (−0.64 to 2.55)	−0.61 (−0.69 to −0.50)	−0.08 (−0.69 to 1.99)	−0.65 (−0.88 to 0.10)	1.59 (−0.11 to 7.34)	0.17 (−0.60 to 2.71)	−0.16 (−0.72 to 1.74)	−0.69 (−0.90 to 0)

ASR. Age-standardized rate; DALYs. Disability-adjusted life years; YLLs. Years of life lost; YLDs. Years lived with disability

Table S5 Average annual percentage change (AAPC) of ASMR and ASDR attributable to tobacco use, by sex and tobacco type from 1990 to 2023

**Risk factor**	**AAPC**					
	**Deaths (%, 95% CI)**	***t***	***P*-value**	**DALYs (%, 95% CI)**	***t***	***P*-value**
Tobacco						
Both	−2.34 (−2.74 to −1.94)	−11.30	<0.001	−2.53 (−2.91 to −2.14)	−12.78	<0.001
Male	−3.02 (−3.29 to −2.76)	−21.88	<0.001	−3.41 (−3.62 to −3.21)	−32.07	<0.001
Female	−2.24 (−2.73 to −1.75)	−8.91	<0.001	−2.31 (−2.73 to −1.90)	−10.78	<0.001
Smoking						
Both	−2.68 (−3.05 to −2.32)	−14.26	<0.001	−3.5 (−3.80 to −3.20)	−22.56	<0.001
Male	−2.8 (−3.08 to −2.53)	−19.74	<0.001	−3.49 (−3.74 to −3.25)	−27.27	<0.001
Female	−2.59 (−2.98 to −2.20)	−12.81	<0.001	−3.51 (−3.88 to −3.14)	−18.31	<0.001
Secondhand smoke						
Both	−3.5 (−3.80 to −3.20)	−14.26	<0.001	−2.68 (−3.05 to −2.32)	−22.56	<0.001
Male	−3.49 (−3.74 to −3.25)	−19.74	<0.001	−2.8 (−3.08 to −2.53)	−27.27	<0.001
Female	−3.51 (−3.88 to −3.14)	−12.81	<0.001	−2.59 (−2.98 to −2.20)	−18.31	<0.001
Chewing tobacco						
Both	−2.02 (−2.46 to −1.58)	−8.93	<0.001	−2.05 (−2.50 to −1.60)	−8.82	<0.001
Male	−3.05 (−3.26 to −2.84)	−7.93	<0.001	−3.14 (−3.35 to −2.92)	−7.82	<0.001
Female	−1.67 (−2.19 to −1.14)	−6.93	<0.001	−1.71 (−2.25 to −1.18)	−6.82	<0.001

ASMR. Age-standardized mortality rate; ASDR. Age-standardized disability-adjusted life year rate

Table S6 Top 10 leading specific causes of death and DALYs attributable to tobacco in China in 2023

Rank	Death		DALYs	
	**Cause**	**Proportion (%)**	**Cause**	**Proportion (%)**
1	Tracheal, bronchus, and lung cancer	22.18	Tracheal, bronchus, and lung cancer	21.08
2	Ischemic heart disease	20.73	Ischemic heart disease	19.58
3	Chronic obstructive pulmonary disease	20.52	Stroke	17.82
4	Stroke	17.35	Chronic obstructive pulmonary disease	15.32
5	Esophageal cancer	5.29	Esophageal cancer	5.00
6	Stomach cancer	2.61	Diabetes mellitus	3.17
7	Lower respiratory infections	1.86	Low back pain	3.00
8	Alzheimer's disease and other dementias	1.21	Stomach cancer	2.52
9	Diabetes mellitus	1.05	Lower respiratory infections	1.55
10	Pancreatic cancer	0.96	Alzheimer's disease and other dementias	1.20

DALY. Disability-adjusted life year

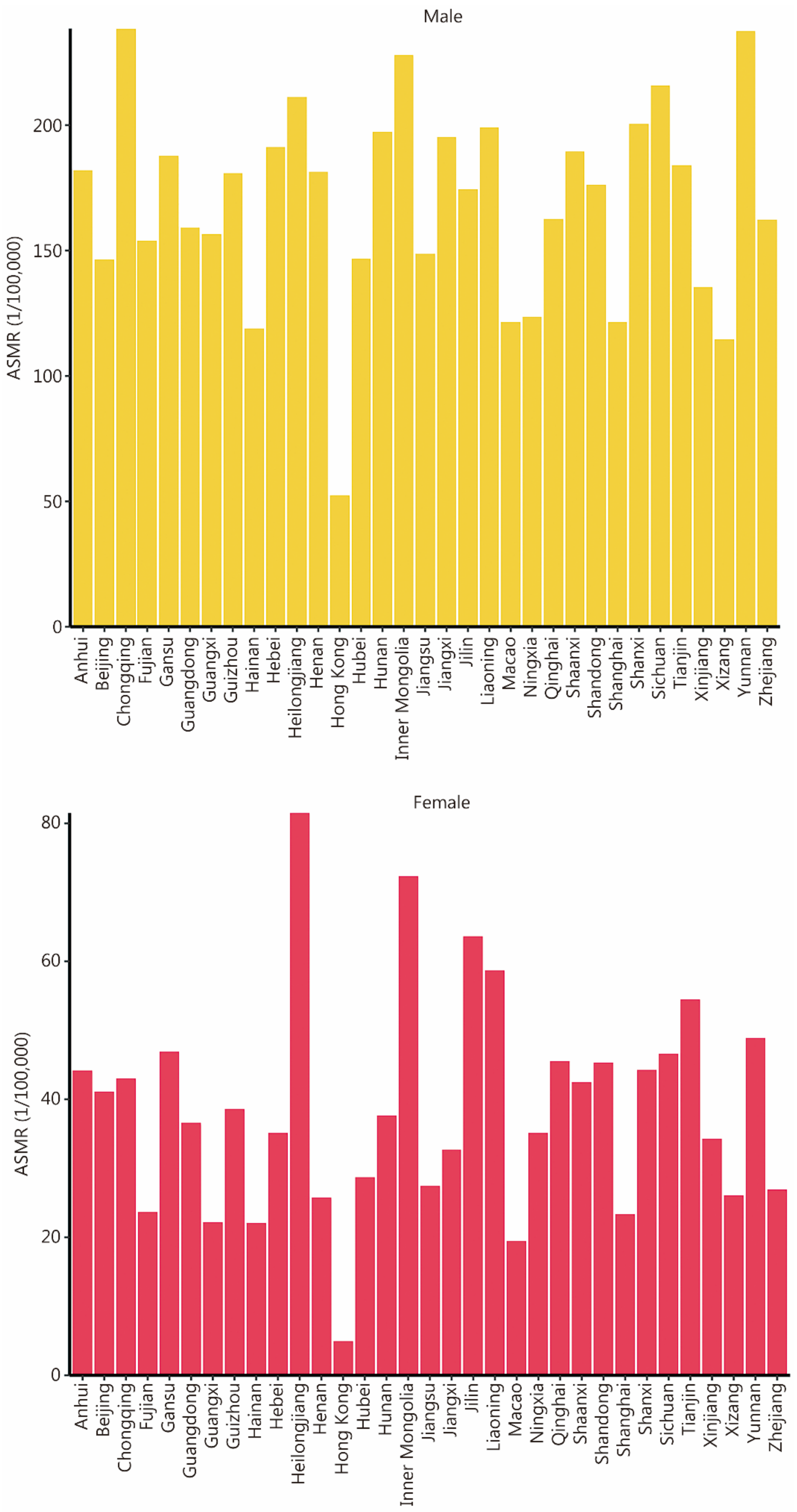

Fig. S1 ASMR attributable to tobacco use among males and females across provinces in 2023. ASMR. Age-standardized mortality rate

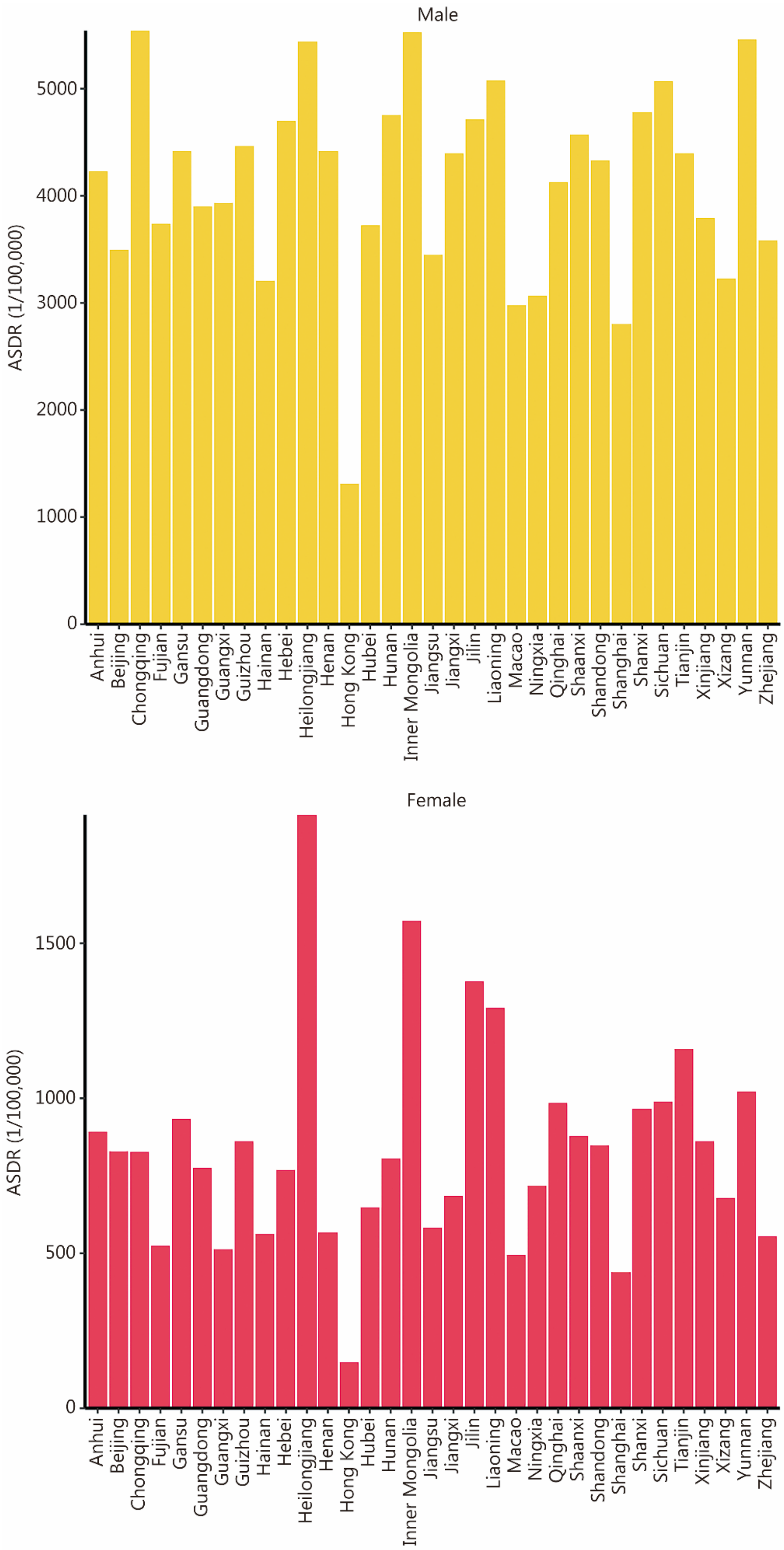

Fig. S2 ASDR attributable to tobacco use among males and females across provinces in 2023. ASDR. Age-standardized disability-adjusted life year rate
